# Editorial: Architects of Endocrine-Related Tumour Growth

**DOI:** 10.3389/fendo.2022.1046478

**Published:** 2022-10-24

**Authors:** María Eugenia Sabatino, Graciela Díaz-Torga, Ana Lucía De Paul

**Affiliations:** ^1^ Instituto de Ciencia y Tecnología de Alimentos Córdoba -Consejo Nacional de Investigaciones Científicas y Técnicas (ICyTAC-CONICET), Córdoba, Argentina; ^2^ Instituto de Biología y Medicina Experimental (IBYME), Consejo Nacional de Investigaciones Científicas y Técnicas (CONICET), Buenos Aires, Argentina; ^3^ Consejo Nacional de Investigaciones Científicas y Técnicas (CONICET), Instituto de Investigación en Ciencias de la Salud (INICSA), Córdoba, Argentina; ^4^ Universidad Nacional de Córdoba, Facultad de Ciencias Médicas, Centro de Microscopía Electrónica, Córdoba, Argentina

**Keywords:** endocrine tumour, cellular fates, proliferation, cellular death, tumour growth regulation

In contrast with other kinds of neoplasia, endocrine tumours are less frequently recorded, in part because many of these go undiagnosed. As endocrine tissues usually present several subtypes of cells, differentiation between normal and tumour tissue is difficult, as complex phenotypes and often paradoxical behaviour patterns are involved. This makes a reliable prognosis and successful management of endocrine tumours difficult to achieve. Hence, this situation has led to a relatively slow improvement in establishing the pathogenesis of endocrine tumours. The present Research Topic “Architects of Endocrine-Related Tumour Growth” focuses on the nature of the heterogeneous cellular features and pathways involved in the cellular growth programs that shape tumour development and considers their implications in the evolution and treatment of endocrine tumours. The involvement of inextricable signalling networks is addressed, in which the orchestration of many interdependent pathways and cellular fates may guide, preserve, or interfere with endocrine-related tumour growth.

A current approach for signalling network determination is presented by Zhu et al. This group develop a prediction model for testicular germ cell tumour metastasis and recurrence based on an mRNAlncRNA-miRNA regulatory network constructed from differentially expressed RNA profiles among tumoral and normal tissue, and integrated with RNA-Seq data and public clinical databases. They also describe the correlation between the dysregulation of mRNA with immunomodulation, cell migration and invasion, indicating a link between immune cell infiltration and testicular germ cell tumours. Finally, they identify GRK4, PCYT2, and RGSL1A as being potential specific biomarkers that could predict relapse-free survival, tumour immunity and chemotherapeutic resistance.

With the aim of improving early diagnosis of tumour growth, an original article by Guang et al. explores the ultrasonic features of preoperative clinical data combined with superb microvascular and contrast-enhanced ultrasound imaging of thyroid nodules and papillary thyroid cancer (PTC). These authors perform a multi-factor analysis to determine the predictive value of central lymph node metastasis from PTC. This provides a correlation between metastatic behaviour and ultrasound characteristics such as tumour size, multifocality, microcalcification particularities, vascularization, and contact with the adjacent capsule. In addition, the study establishes a criterion for a high risk of lymph node metastasis based on the grayscale ultrasound data, thereby contributing to the development of a clinical basis for the early diagnosis, treatment and prognosis of thyroid cancer.

To further the understanding of the intricate molecular dialogs involved in endocrine-related tumour growth, Martin et al. explore the role of nuclear localization of the insulin-like growth factor receptor (IGF1R) in the regulation of glioblastoma cellular behaviour. These authors modify a glioblastoma cell line to either allow or block IGF1R translocation to the nucleus. This study reveals that IGF1R nuclear localization increases cell motility, as well as the metabolic activity of glucose and lipids, without having any repercussions on proliferation. Furthermore, *in vivo* studies using subcutaneous tumour xenografts show that nuclear-localized IGF1R provoke metabolic shifts that result in increased proliferation and accelerated tumour growth. However, it also sensitised cells to pharmacological therapy with IGF1R inhibitors. These findings support the hypothesis that although IGF1R nuclear localization in glioblastoma cells may contribute to an aggressive phenotype, it also represents a potential therapeutic possibility for patients whose tumours exhibit this intracellular localization.

Tumour development requires cellular physiology and metabolic reprogramming to deal with environmental modifications and new cellular demands. Moreover, the dynamical tumour ecosystem may adjust its needs and influence cellular neighbourhoods. In this regard, Sacca and Calvo review the impact of several factors and fatty acids secreted by the periprostatic adipose tissue (PPAT) on the prostate cancer (PCa) microenvironment. In this article, they describe how PPAT acts co-ordinately with PCa development by adjusting its metabolic performance according to the stage of tumour progression. These authors report that in advanced tumours, the metabolic secretome profile is enriched in energy balance and hormone response molecular signalling, with the catabolic metabolism of polyamine, protein, and nucleotide probably required for tumour growth. Thus, these metabolic reprogramming hallmarks of PPAT secretome may serve as indicators for the early diagnosis of high-risk tumours and provide positive therapeutic effects.

Tumour progression represents the coevolution and integration of phenotypic cellular changes within the microenvironment. Ritch and Telleira extensively assess these events for epithelial ovarian cancer (EOC). In this review, they describe the sequence of microenvironments encountered by EOC cells during transcoelomic dissemination, and the special aspects of these scenarios that can influence cellular fates and tumour progression. These authors describe EOC cell fluctuations within the different cellular ecosystems, starting at the ovaries or the fallopian tubes, whose pro-inflammatory surroundings could favour precursor lesions. In addition, the detachment process is followed by the ability to survive in the peritoneal fluid, thereby evading cell death signalling and enabling suitable cellular communication. Metastatic EOC cells require adherence, and migrate as spheroids through the peritoneum, being able to reach distant tissue and invade as primary or secondary tumours. Related to this, the involvement of the microenvironment effects over EOC chemoresistance has been consistently reported. This extensive overview of the entire process allows for a better understanding of the role of cellular and non-cellular factors during EOC transcoelomic dissemination, promoting the creation of novel therapeutic strategies to treat this pathology.

For tumour development to be achievable, cells must acquire new abilities beyond unregulated proliferation. Different cellular outcomes may emerge during tumour development that can either favour or constrain cell growth. Sabatino et al. summarise current findings in pituitary neuroendocrine tumours by examining the range of possible cellular fates involved throughout tumorigenesis. This article emphasises the relevance and importance of integrating emerging fields in pituitary research, such as cellular senescence, autophagy, mitochondrial function, metabolic reprogramming, stem cell niches and non-apoptotic cell death during tumour progression. These authors expose the need for innovative models that are able to reflect the complex interplay that occurs during tumour pathogenesis. This review also highlights the requirement for the incorporation and systematization of newly available data involving tumour origin, behaviour and plausible clinical results, in order to improve tumour classification.

Sousa et al. review existing knowledge about autophagy in adrenal tumours. As previously reported for different tumours, autophagy in this neoplasia presents a dual role towards pro-survival or pro-death, depending on the context, and its action is interconnected with other cellular processes. Both the induction and inhibition of autophagy have revealed possibilities for treatment strategies for adrenal neoplasms, with the impact of autophagy on tumour progression appearing to depend on the degree of tumour evolution and the type of therapy employed. Adrenal autophagy might be associated with steroid synthesis and extracellular vesicle biogenesis, suggesting potential targets for tumour diagnosis and management. These authors emphasise that further research is still required to clarify the clinical implication of autophagy modulation in adrenal tumours.

In conclusion, the evidence presented here shows that the amount of data accumulated over recent decades highlights the complexity of endocrine-related tumorigenic mechanisms. Indeed, the pathogenesis of multiple endocrine neoplasia is still unclear, with their management mostly depending on histopathological criteria. Thus, a proper elucidation of the plethora of diverse genetic, epigenetic, and biochemical signalling pathways, together with a better understanding of the metabolically and physiological cellular outcomes implicated in tumourigenesis, would be particularly useful, as using histological characteristics alone restricts the accuracy of the diagnosis and prognosis. We have now reached a point where it is necessary to create broader modelling strategies that allow us to consider not only a single component, but the sophisticated and multifaceted network that influences tumour progression.

## Author contributions

MES, GD-T and ALD contributed to conception and design of the editorial. MES wrote the first draft of the manuscript. All authors contributed to manuscript revision, read, and approved the submitted version.

## Funding

This work was funded by Consejo Nacional de Investigaciones Científicas y Técnicas (CONICET PIP 2020-2023 No. 11220200102210), Secretaría de Ciencia y Tecnología, Universidad Nacional de Córdoba (SECyT-UNC 2018-2022 Nº33620180100675CB), and Agencia Nacional de Promoción Científica y Tecnológica - Ministerio de Ciencia y Tecnología (FONCYT-PICT 0950-2020) to ALD.

## Conflict of interest

The authors declare that the research was conducted in the absence of any commercial or financial relationships that could be construed as a potential conflict of interest.

## Publisher’s note

All claims expressed in this article are solely those of the authors and do not necessarily represent those of their affiliated organizations, or those of the publisher, the editors and the reviewers. Any product that may be evaluated in this article, or claim that may be made by its manufacturer, is not guaranteed or endorsed by the publisher.

